# Laminectomy for Penetrating Spinal Cord Injury with Retained Foreign Bodies

**DOI:** 10.1111/os.13332

**Published:** 2022-06-09

**Authors:** Peng Zhang, Xiaoyang Liu, Dongsheng Zhou, Qingyu Zhang

**Affiliations:** ^1^ Department of Orthopaedics Shandong Provincial Hospital affiliated to Shandong First Medical University Jinan China

**Keywords:** Laminectomy, Penetrating spinal cord injury, Retained foreign body, Systematic review

## Abstract

**Background:**

Penetrating spinal cord injury (PSCI) with retained foreign bodies (RFB) is rarely observed in clinics and may result in a complete or incomplete neurological deficit. This study was performed to appraise the treatment effect of laminectomy for PSCI with RFB.

**Case Presentation:**

This study presented three patients referred to a tertiary hospital between August 2011 and October 2018 due to PSCI with RFB and receiving laminectomy. The first patient was a 25‐year‐old female with a butcher's knife piercing the T_9_ lamina and T_10_ vertebral body obliquely; the second was a 49‐year‐old male who suffered a perforating wound of the cervical spinal canal and injury of vertebral artery from foreign glass, while the third was a 60‐year‐old male with a wooden stick penetrating stomach and terminating in the L_1_ lamina. The first and second patients immediately underwent laminectomy for debridement and removal of RFB, while the third received two‐staged operations to remove the retained stick thoroughly. Unfortunately cases 1 and 3 eventually resulted in total paralysis and case 2 revealed no improvement in myodynamia. Then, Medline/PubMed, Embase and the Cochrane Library were systematically searched, and 23 articles involving 25 additional cases with this kind of injury were included for analysis.

**Conclusions:**

The optimal treatment strategy for penetrating spinal cord injury with retained foreign bodies remains challenging and should be assessed case‐by‐case. If possible, surgical removal of foreign bodies by laminectomy is preferred immediately to prevent delayed presentation and persistent contamination. Meanwhile, a multidisciplinary team is needed to address concomitant injuries.

## Graphical abstract

In this study, we presented three patients referred to a tertiary hospital due to penetrating spinal cord injuries with retained foreign bodies and conducted a systematic review on this topic.

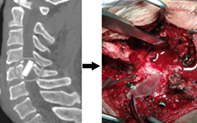

## Introduction

The overwhelming majority of spinal cord injuries cases are due to blunt trauma from falls or traffic accidents, and those secondary to penetrating injury are rarely observed in the clinical setting.^1^ Penetrating spinal cord injury (PSCI) disproportionately affects young males in their 20s and 30s, and may result in severe neurological deficits.^2^, ^3^ Up to now, most of our knowledge and experience about PSCI comes from several large case series from South Africa published in the 1960–70s.^2^, ^3^ When referring to the emergency room, those with retained foreign bodies (RFB) are even rarer. These foreign bodies can enter the spinal canal *via* the interlaminar space or through the intervertebral foramen and lodge in the vertebral bodies, laminae, or pedicles,^4^, ^5^ posing a tough challenge for surgeons about the initial care of the patient, evaluation of the potentially affected structures and the appropriate sequence of therapeutic interventions.

Although multiple case series have been published, there is still controversy in the management paradigm for PSCI with RFB. Laminectomy is usually indicated to facilitate the complete removal of foreign bodies lodged in the spinal canal. This article reported three cases of PSCI with RFB in a tertiary hospital manifesting incomplete or complete neurological deficits and receiving laminectomy. Meanwhile, a systematic review of relevant literature was provided to summarize the clinical characteristics of PSCI with RFB and appraise the treatment effect of laminectomy. We hope that this case report and systematic review will help clinicians to identify the optimal treatment regimen for this kind of injury and improve therapeutic efficacy.

## Case Presentation

### Case 1

**Fig. 1:**
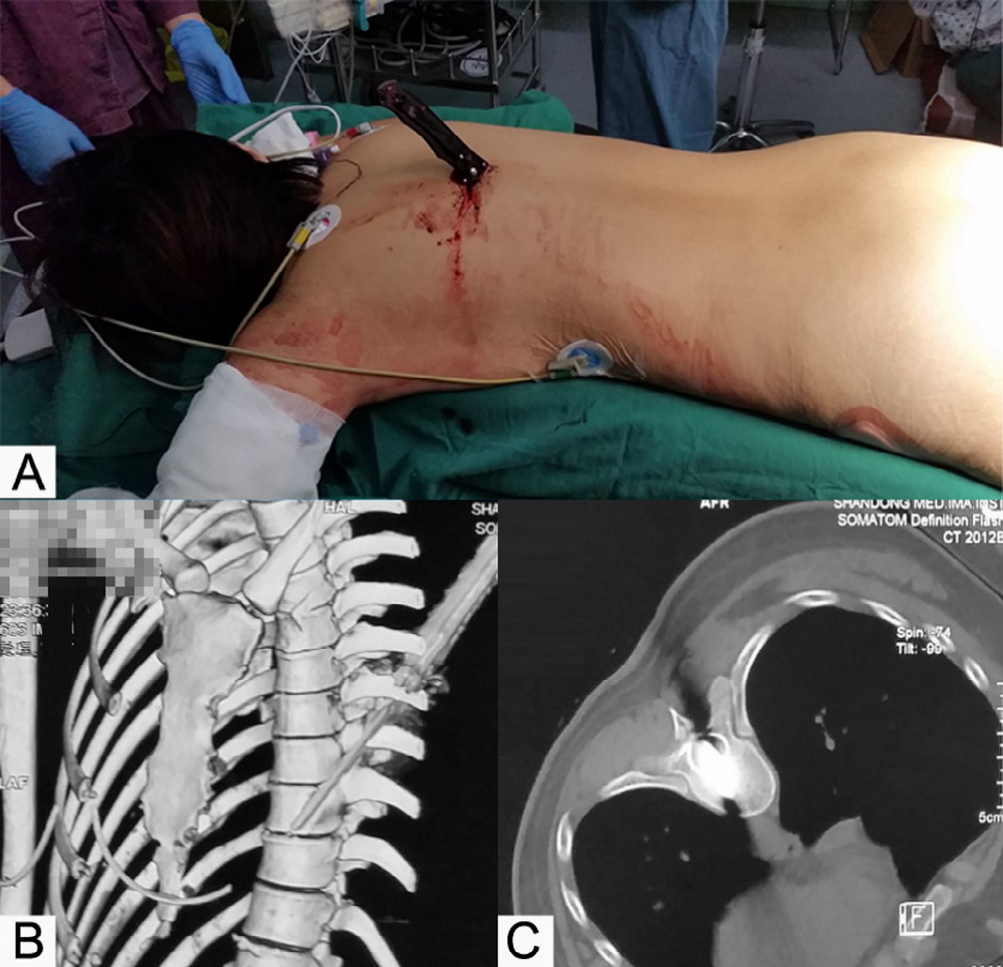
Case 1 (25‐year‐old female). (A) the patient was assaulted with a sharp knife; (B) three‐dimensional CT reconstruction and (C) coronal CT revealed a foreign body extending through the spinal canal and terminating in the thoracic cavity

A 25‐year‐old female was stabbed with a sharp knife in the lower back during an altercation. On admission, a knife was obliquely plugged in the left paraspinal region at the thoracic level with the handle sticking out (Figure 1(A)). Physical examination revealed apparent paralysis similar to Brown–Séquard syndrome: loss of myodynamia in the left leg, muscular weakness and loss of sensation to a pinprick in the right leg, as well as bowel and bladder incontinence, which rendered an American Spinal Injury Association (ASIA) score of C. CT revealed that a 10 cm hyper‐dense foreign body piercing the right T_9_ lamina, traversed the thoracic spinal cord and penetrated the T_10_ vertebral body with the tip terminating in the left chest cavity (Figure 1(B),(C)).

After general anesthesia, the course taken by the knife was traced. The blade penetrated the left paraspinal muscles, pierced the interspinous ligaments and entered the T_9_ vertebral body through its left pedicle. To obtain sufficient surgical vision, subperiosteal dissection and subsequent drilling were performed around the insertion point of the knife. Laminectomy of the T_9_‐T_10_ vertebrae was performed and it could be noticed that the blade transected the left part of the thoracic spinal cord. The knife was removed smoothly under direct vision to maintain its trajectory and then the lacerated spinal dura mater was carefully sutured. Fluoroscopy verified that there was no residual debris lodging within the spine, the incision was sutured layer by layer. Besides the routine drainage tube, a chest drainage tube was placed by a cardiothoracic surgeon to address the hemothorax.

The patient tolerated her surgery well and was followed up clinically 3 years after injury. No complications were observed but unfortunately this patient lapsed to total paraplegia ultimately (ASIA score of A).

### Case 2

**Fig. 2:**
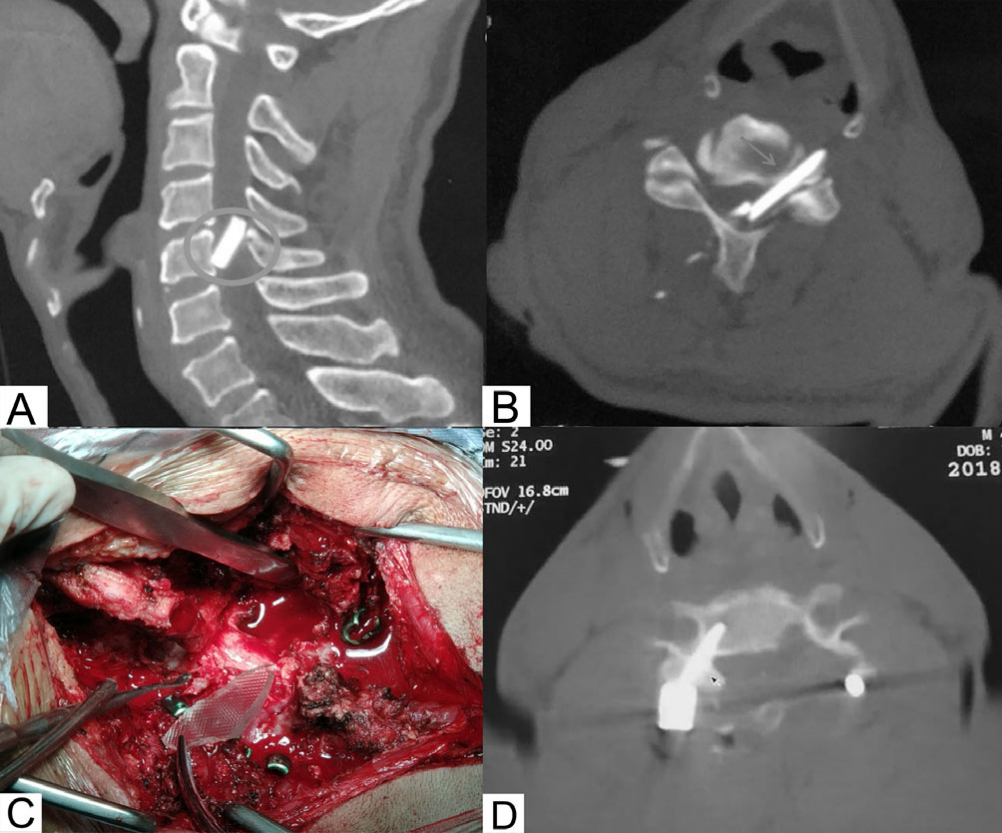
Case 2 (49‐year‐old male). (A) and (B) CT of the cervical spine indicated that foreign glass entered the spinal canal at the level of C_4_; (C) after removing the foreign glass, no more hemorrhage was induced and (D) laminectomy and internal fixation with lateral mass screws

A 49‐year‐old male suffered a posteriorly penetrating injury to his cervical spine after falling from a window. The myodynamia of the right upper limb was grade 3/5; in the left upper and both lower limbs, the myodynamia was grade 0/5, while his bowel and bladder function remained intact. CT of the cervical spine indicated that foreign glass penetrated the right side of the spinous process with a left and downward trajectory and entered the spinal canal at the C_4_ level (Figure 2(A),(B)). CT angiography (CTA) revealed incomplete occlusion of the left vertebral artery at the C_5_ level.

The therapeutic plan included endovascular repair of potential vertebral artery injury, stabilization of cervical vertebrae, and removal of the foreign glass. The posterior midline approach was taken to expose the bilateral C_2_‐C_6_ laminae. C_4_‐C_5_ laminectomy was performed to make it easier to retrieve the glass and exploration revealed that the spinal cord was locally lacerated. The posterior wall of the C_4_‐C_5_ left transverse foramen was excised to expose the vertebral artery. No hematoma or active bleeding surrounding the vertebral artery was observed and after removing the foreign glass, no more hemorrhage was induced (Figure 2(C)). Meanwhile, a small piece of bone block was found to have been separated from the C_5_ vertebra by the foreign glass. Both the bone block and glass crushed the vertebral artery in the foramen transversarium of C_5_. After confirming that there was no compression for the spinal cord, the lacerated spinal dura matter was repaired and internal fixation with lateral mass screws was carried out (Figure 2(D)).

The patient had an uncomplicated hospital stay and was discharged at 14 days postoperatively. However, at the 6‐month postoperative follow‐up, no recovery of myodynamia or sensation were observed (ASIA score of C).

### Case 3

**Fig. 3:**
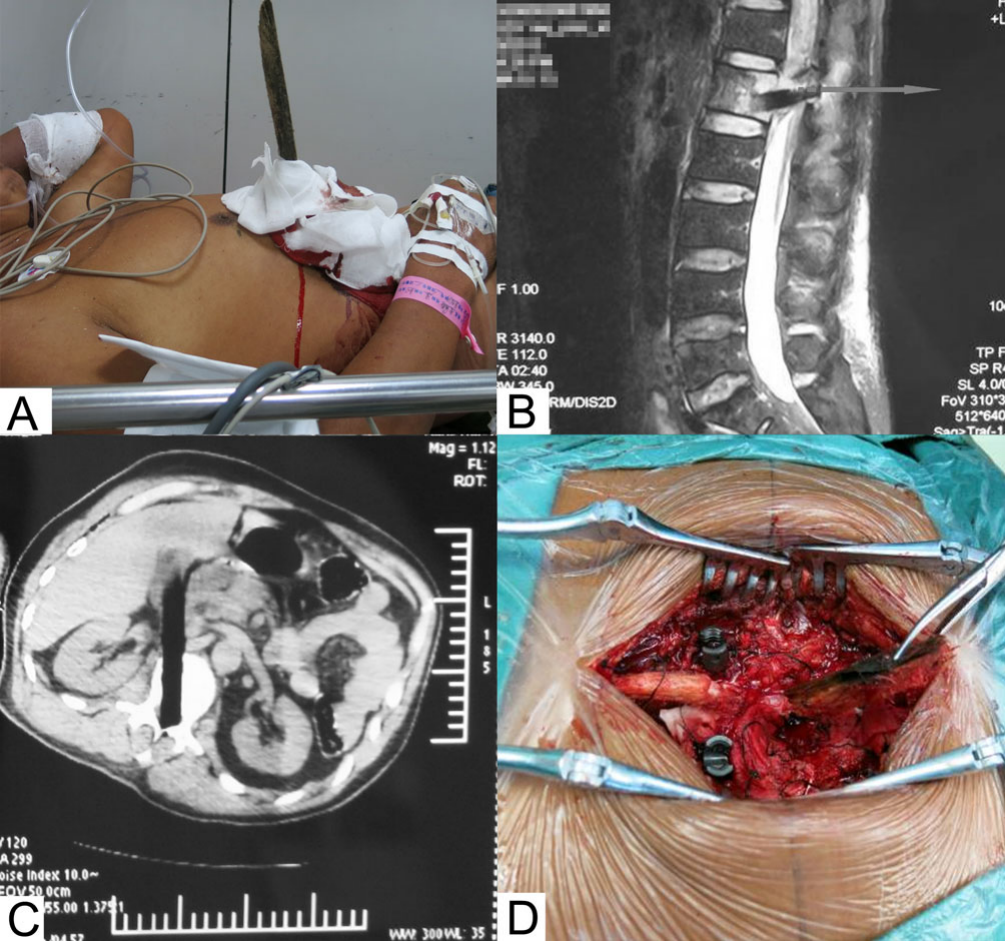
Case 3 (60‐year‐old male). **(**A) the patient presented with a penetrating injury from a wooden stick; (B) sagittal MRI demonstrated a foreign body penetrating the L_1_ vertebra and spinal canal; (C) coronal computed tomography revealed a foreign body extending through the liver, inferior vena cava, kidney and L_1_ vertebra and (D) laminectomy and internal fixation with lateral mass screws

When a 60‐year‐old man was sawing wood, a wedge‐shaped wooden stick flew out and penetrated his body from the right lower abdomen (Figure 3(A)). Sensation and motor functions in both lower extremities were completely lost, rendering an ASIA score of A. MRI and CT scan revealed an injury to the left lobe of the liver, inferior vena cava, left kidney and the L1 vertebra (Figure 3(B),(C)).

Immediately after admission, he underwent laparotomy through an oblique incision following the costal margin on the right side. After the hematocele in the abdominal cavity was cleared, it was seen that a wooden stick penetrated the chest wall, entered the liver through the left inner lobe, and exited the liver from the calot's triangle; subsequently, the stick pierced the inferior vena cava and the L_1_ vertebra. The wooden stick was first cut along the chest wall; then the inferior vena cava was blocked, and the rest of the stick was pulled out. Prolene sutures were used to suture the inferior vena cava and liver fissures.

After the first operation, the patient accepted conservative treatment for 3 weeks. During the second procedure, a posterior midline incision was performed, centering at the level of the L_1_ vertebra. It was noticed that there was a residual part of the wooden stick coming up from the interlaminar space between L_1_‐L_2_. After the pedicle screws were inserted on both sides of the T_12_ and L_2_ vertebrae, the spinous process and lamina of the L_1_ vertebra were excised to expose the spinal canal. It could be seen that the wooden stick penetrated the spinal cord cone and L_1_ vertebral body, rendering contusions of the cauda equina and dural sac. Then the stick was removed smoothly and the surrounding wood chips were cleaned up (Figure 3(D)). After thorough irrigation, the lacerated spinal dura mater was sutured and the spinal rods were firmly fixed. However, postoperative follow‐up for 3 years revealed no recovery of the lower extremity paralysis. The patient maintained an ASIA score of A.

## Systematic Review and Discussion

According to the PRISMA statement,^6^ three electronic databases (PubMed, Embase, and the Cochrane Library) were searched for entries recorded from the time of database inception to March 1, 2021, using a combination of keywords including “penetrating spinal injury,” “retained foreign body” and “laminectomy.” Meanwhile, bibliographies of relevant articles were also hand‐screened to retrieve additional records. Studies included in the systematic review need to meet the following criteria: (i) participants, patients admitted for PCSI with RFB; (ii) intervention, laminectomy; (iii) outcome, adequate data could be extracted to assess the prognosis; and (iv) study design, case reports or case series. Requisite data were extracted and recorded to standardized excel files.

**Fig. 4:**
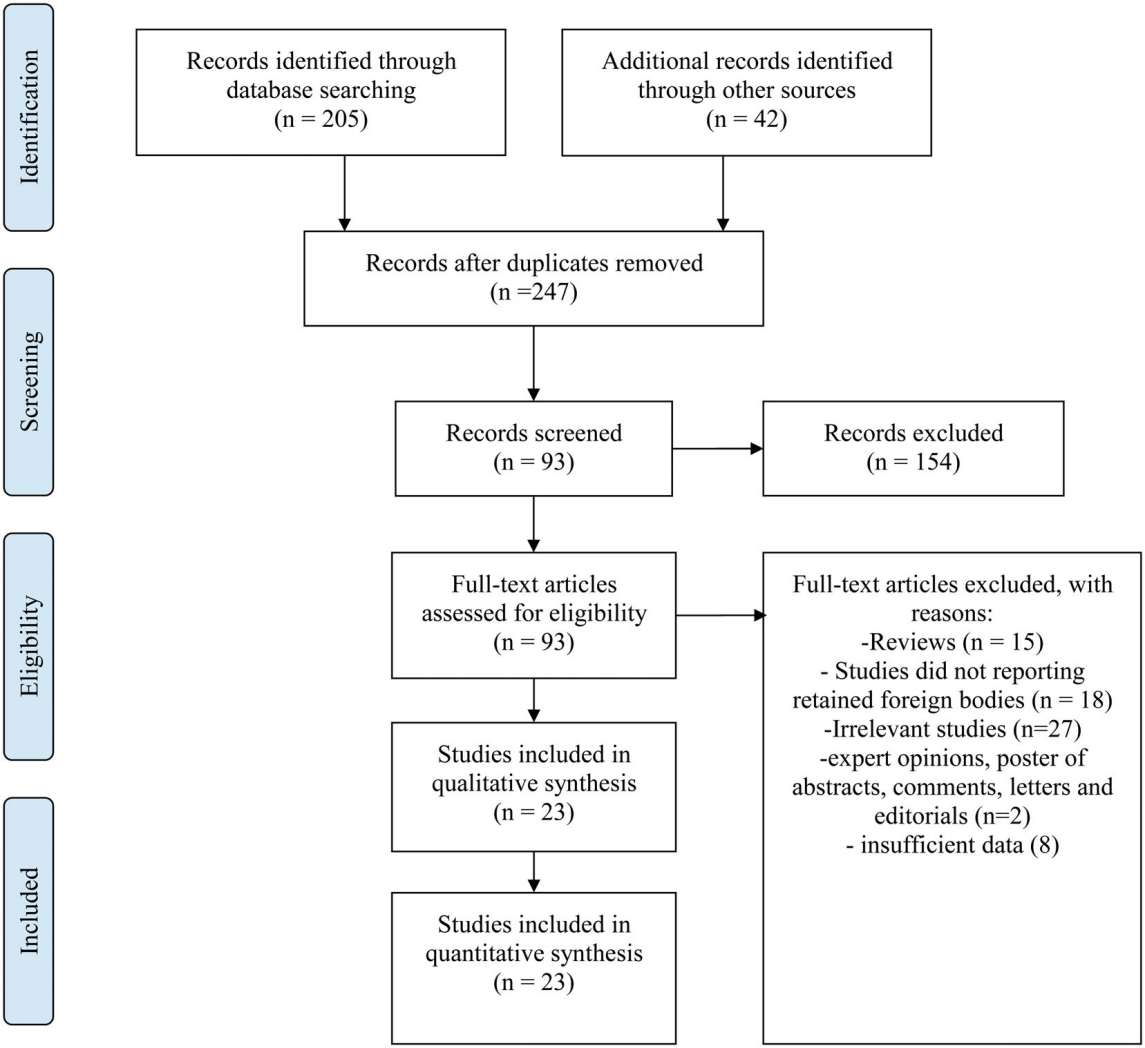
Diagram for study selection process

Eventually, 23 articles^7^, ^8^, ^9^, ^10^, ^11^, ^12^, ^13^, ^14^, ^15^, ^16^, ^17^, ^18^, ^19^, ^20^, ^21^, ^22^, ^23^, ^24^, ^25^, ^26^, ^27^, ^28^, ^29^ published between 1950 to 2021 involving 25 patients were included. Plus three cases presented in the current article, 28 patients with this kind of injury were analyzed (Table 1). The search flow diagram is shown in Figure 4. The included patients' ages ranged from 11 to 74 (mean 38.9) with a male prevalence of 85.7% (24/28), consistent with previous observations that PCSI mainly affects young males in their 20s and 30s. The most frequent clinical presentation was lower limb paresis and the most commonly seen RFB were knife blades. Six patients have an injury at the cervical level, 16 at the thoracic level and six at the lumbar level. Ten patients developed delayed presentations with an interval between initial injury and onset of symptoms ranging from 1 day to 21 years. Two patients remained neurologically intact. All patients received laminectomy to remove the RFB and 67.86% (19/28) of them revealed postoperative improvement of neurologic symptoms. One patient died eventually at the end of the follow‐up.

**TABLE 1:** Characteristics of included studies

Author	Year	Country	Gender	Age of injury	Level	Foreign body	Time to symptoms	Presentation	Outcome
Zhang *et al*.^1^	2021	China	F	25	T_10_	Knife blade	0	Bss	No Improvement
Zhang *et al*.^2^	2021	China	M	49	C_4_‐C_5_	Glass	0	Paresis	No Improvement
Zhang *et al*.^3^	2021	China	M	60	L_1_	Wood stick	0	Paresis and Paralysis	No Improvement
Ojukwu *et al*.^7^	2021	USA	M	34	L_1_‐L_2_	Bullet	0	Paraplegia without Sphincter Function	No Improvement
Yoneoka *et al*.^8^	2020	Japan	M	75	C_7_	Glass	0	None	No Sequelae
Takagi *et al*.^9^	2020	Japan	M	47	C1	Metal wire	0	Left Paresis	Improved
Moldovan et a.^10^	2019	USA	M	49	T_7_	Scissor	0	Pain	No Sequelae
Onishi *et al*.^12^	2017	Brazil	M	31	C_3_‐C_4_	Plant needle	4 yrs.	Progressive Spastic Quadriparesis	Improved
Chen *et al*.^11^	2018	China	M	60	T_4_‐T_5_	Nail	NA	Back Pain	Improved
Komarowska *et al*.^13^	2013	Poland	F	11	T_11_‐T_12_	Glass	0	Paresis and Paralysis	No Improvement
Cheng *et al*.^14^	2012	USA	M	21	C_2_‐C_3_	Bullet	0	Paresis	Improved
Li *et al*.^15^	2012	USA	M	17	T_7_‐T_8_	Knife blade	0	None	No Sequelae
Ye *et al*.^16^	2010	China	M	54	T_5_	Iron fence	0	Bss	Improved
Dogan *et al*.^18^	2008	Turkey	M	22	T_12_	Knife blade	0	Monoparesis	Improved
Russell *et al*.^17^	2009	Australia	M	41	C_5_‐C_6_	Power drill	0	Bss	Improved
Groen *et al*.^19^	2002	Netherlands	M	17	T_6_‐T_9_	Stingray spine	0	Paraparesis	Improved
Manzone *et al*.^20^	2001	Argentina	M	22	T_4_‐T_5_	Knife blade	0	Paraparesis	Improved
Kulkarni *et al*.^21^	2000	Canada	M	31	T_11_‐T_12_	Knife blade	4 weeks	Right Leg Numb and Weakness	No Improvement
Tokushige *et al*.^22^	2000	Japan	M	66	L_4_‐S_1_	Stell rod	0	Right Leg Paralysis	Died
			M	29	L_1_	Stell rod	0	None	Improved
Fung *et al*.^23^	1992	China	M	53	T_2_	Scissor	15 yrs.	Left Leg Paralysis	Improved
Criado *et al*.^24^	1990	USA	M	21	T_7_	Knife blade	3 days	Back Pain	No Improvement
Wu *et al*.^25^	1986	USA	F	65	L_1_‐T_2_	Bullet	18 yrs.	Pain In the Lower Back and Leg	Improved
			M	24	L_3_‐T_4_	Glass	1 day	Right Leg Paralysis	No Improvement
Ehni *et al*.^26^	1983	USA	F	41	T_8_	Scissors	Unknown	Spastic Paraparesis	No Improvement
Jones *et al*.^27^	1981	USA	M	46	T_5_‐T_6_	Knife blade	8 yrs.	Left Leg Paralysis	Improved
Wolf *et al*.^28^	1973	USA	M	35	T_6_	Knife blade	21 yrs.	Spastic Paraparesis	Improved
Castillo *et al*.^29^	1950	USA	M	43	T_4_	Knife blade	19 yrs.	Monoparesis	Improved

Abbreviation: Bss, Brown‐Séquard syndrome.

According to Lipschitz *et al*.’s study^2^ of 130 patients with non‐missile PSCI, the proportion of lodged weapons was deficient, but in a more recent case series^4^ published in 2015, this rate reached up to 49% (51/104). The discrepancy could be explained by the advancement of imaging techniques.^2^, ^5^ In the most extensive series on this topic reported by Peacock *et al*.,^3^ the location of injury was most frequently at the upper thoracic (54–74%) or lower cervical (18–30%) level; only 7–8% involved the lumbosacral area. Meanwhile, concomitant pneumothoraxes and artery injuries were observed in nearly 16% cases.^4^ In the current article, besides the spinal cord, RFB resulted in injuries to the thorax, liver, kidney and inferior vena cava. The manifestation of PSCI with RFB may be presented in an acute or delayed way. After PSCI, most patients immediately exhibit various degrees of neurological deficits, the most common being Brown–Séquard syndrome (45–55%).^3^, ^30^, ^31^ Meanwhile, delayed manifestations could happen from 1 day to 21 years after the initial injury.^28^, ^32^ In most of these cases, RFB failed to be spotted immediately due to inadequate imaging, and a minor trauma might lead to migration of RFB or disruption of previously formed scar tissue adjacent to the spinal cord and/or nerve roots.^25^, ^26^

Following stab wounds, these patients were managed according to the Advanced Trauma Life Support (ATLS) principles.^33^ A complete physical examination involving myodynamia, muscular tension, reflexes and sensation is necessary. Careful consideration of PSCI must be made using X‐ray and CT to identify the number, location and trajectory of lodged fragments.^34^ If a delayed neurological deficit occurs, patients should be overhauled to determine whether there is a residual foreign body or a new onset of operation‐related complications. For case 3, a critical step was to prevent hemorrhage upon extraction of the foreign body. The vascular injury could be ruled out with CTA. Spinal MRI is not routinely performed due to the possibility of *in situ* ferromagnetic materials but could be used if there is evidence of neurological deterioration, spinal abscess, empyema, hemorrhage or epidural hematoma after removing the blade.

Immediate removal of the RFB and decompression are indicated, and the surgical options include laminectomy and minimally invasive fluoroscopic technique.^10^ The latter was associated with minor damage, shorter hospital stays as well as speedier rehabilitation, but also a lower chance to achieve thorough debridement. Until now, decompressive laminectomy and watertight closure of spinal dura mater remain the mainstream choice for these cases.^4^, ^10^ For patients suffering PSCI with RFB, it is advisable to promptly initiate broad‐spectrum antibiotics until the lumbar drain is removed and there is no concern for CSF leakage.^35^ Meanwhile, glucocorticoid use is now not recommended for its adverse outcome and limited benefits.^4^, ^36^ Conservative methods such as fluid infusion, blood transfusion and physiotherapy are important components of the treatment regimen. In Lipschitz *et al*.’s case series,^2^ only 4.6% of patients improved clinically, while In Enicker *et al*.’s report,^4^ none of those ASIA A or B grade cases showed clinical improvement and only four patients with incomplete SCI recovered to ASIA E status. As to the three patients admitted to our center, cases 1 and 3 were categorized as ASIA C and A grade respectively after initial injury but unfortunately, both ended with ASIA A grade. Meanwhile, case 2 revealed no improvement in myodynamia after the operation. A surprisingly satisfactory recovery (67.86%) of laminectomy was achieved for the case reports included in the systematic review, possibly due to a publication bias.

### Conclusion

The optimal treatment strategy for penetrating spinal cord injury with retained foreign bodies remains challenging for surgeons and should be assessed case‐by‐case. If possible, surgical removal of foreign bodies by laminectomy is preferred immediately to prevent delayed presentation and persistent contamination. Meanwhile, a multidisciplinary team is needed to address concomitant injuries.

## Author Contributions

All authors have read and approved the manuscript. Each author has contributed individually and significantly to the development of the manuscript. P.Z. was the main contributor to drafting the manuscript. X.L. and D.Z. performed literature search and analysis. Q.Z. designed the study, performed the manuscript review and contributed to the intellectual content of the study.

## Declaration

All authors listed meet the authorship criteria according to the latest guidelines of the International Committee of Medical Journal Editors and all authors are in agreement with the manuscript.
